# Community’s extent of use and approval of extended pharmacy services in community pharmacies in Southwest Ethiopia

**DOI:** 10.1371/journal.pone.0230863

**Published:** 2020-04-02

**Authors:** Zelalem Tilahun Tesfaye, Malede Berihun Yismaw

**Affiliations:** 1 Department of Clinical Pharmacy, School of Pharmacy, College of Medicine and Health Sciences, University of Gondar, Gondar, Ethiopia; 2 Department of Pharmacology and Clinical Pharmacy, School of Pharmacy, College of Health Sciences, Addis Ababa University, Addis Ababa, Ethiopia; KEMRI Wellcome Trust Research Programme, KENYA

## Abstract

**Background:**

The emergence of chronic diseases as major causes of disability and death has necessitated the introduction of new strategies to effectively address the ever-changing nature of public health problems. As a result, the role of community pharmacies in promoting public health is growing in recent years through the provision of extended pharmacy services. This study was conducted with the aim of assessing community’s extent of use and approval of extended pharmacy services at community pharmacies in Bonga town, Southwest Ethiopia.

**Materials and methods:**

Community based cross-sectional study was conducted in Bonga town, Southwest Ethiopia, on households selected by systematic random sampling. Data was collected using semi-structured questionnaire. Data was collected by personally delivering questionnaires to respondents in selected households. Results of the study were described by frequency, mean and standard deviation (SD). Binary logistic analysis was performed to identify potential associations between dependent and independent variables.

**Results:**

Out of 356 individuals included in the study, 58.4% recalled visiting community pharmacy premises during the previous six months. Out of these, 34.6% visited the community pharmacies to get extended pharmacy services. College educated participants were 19.4 times more likely to have used extended pharmacy services as compared to illiterate individuals whereas those who earn monthly income more than 5000 Ethiopian Birr were 3.6 times more likely than those with monthly income of 2000 Ethiopian Birr or less. Of the total participants, 91.3% approved the provision of extended pharmacy services in community pharmacies.

**Conclusion:**

The extent of community’s use of extended pharmacy services at community pharmacies was found to be low. Nevertheless, majority of the study subjects approved the provision of extended pharmacy services at community pharmacies. Efforts to improve the practice of extended pharmacy service provision at community pharmacies should be made by all stake holders.

## Introduction

Community pharmacies are places where medicines are stored and dispensed or supplied. They are often alternately referred to as retail drug outlets or retail pharmacies. In this article “community pharmacy” has been used to refer to retail pharmacies and drugstores [1]. Pharmacists working in community pharmacies constitute a significant portion of the healthcare workforce. The emergence of chronic diseases as major causes of disability and death has necessitated the introduction of new strategies to effectively address the ever-changing nature of public health problems. As a result, the role of community pharmacies in promoting public health is growing in recent years through the provision of extended pharmacy services [2, 3].

Extended pharmacy services are healthcare related services provided in pharmacies in addition to the routine dispensing of over-the-counter (OTC) and prescribed medications [4]. Some of healthcare services that could be delivered in community pharmacy settings include health promotion, preventing the occurrence of diseases (e.g. smoking cessation, immunization), responding to symptoms, identifying and referring ill individuals via screening and maintaining health of those with chronic diseases such as diabetes, hypertension. The benefits of pharmacy services in these important public-health issues have been documented in many studies [5–9].

Mortality and morbidity caused by non-communicable diseases can be reduced significantly by routine screening and proper management of chronic diseases, a service which is known to be effectively provided at community pharmacies [10, 11]. As non-communicable diseases have emerged as important public health problem in Ethiopia recently, providing chronic disease screening and management services for the public at community pharmacies will lead to improved outcomes in tackling the problem [12].

Moreover, Ethiopia is one of the countries that fall below the World Health Organization (WHO) threshold of 2.3 healthcare professionals per 1000 people [13]. With such health professionals’ shortage, it would be difficult to meet short-term and long-term healthcare goals set by the country as well as WHO. Extending the role of pharmacists in community pharmacies, however, could contribute to improvement of this shortage by providing skilled professionals in the area of health promotion, disease screening and prevention [14].

The involvement of community pharmacists in the public health roles can also contribute to cost savings on the healthcare system. A report from an Australian study revealed that well-trained pharmacists could reduce healthcare cost by as much as six times the baseline [15]. Consequently, the extended roles of the community pharmacists in the multidisciplinary provision of healthcare have been acknowledged in many developed countries [15–17]. Reduction of cost by engaging pharmacists in certain public health services will have massive impact in developing countries like Ethiopia, where resource scarcity is the major factor that hinders provision of adequate healthcare services [14].

There has been increased interest in broadening the role of community pharmacists in the country’s healthcare system recently. As a result, although at a low level, provision of some healthcare services at community pharmacies and encouraging community engagement in seeking these services had been reported in Addis Ababa, the capital of Ethiopia [18]. However, there is no data regarding the extent of provision of extended services elsewhere in the country, despite these services are much needed in areas with relatively lower accessibility of other healthcare facilities. This study was conducted with the aim of assessing community’s extent of use and approval of extended pharmacy services in community pharmacies.

## Material and methods

The study was conducted in Bonga town, Southwest Ethiopia. The town is located 460 kilometers southwest of the capital Addis Ababa. The study was conducted from July 17, 2017 to August 28, 2017.

Community based cross-sectional study was conducted on households selected by systematic random sampling. Sample size was estimated using single population proportion formula: n=Zα22p(1−p)d2 [19] by using the following assumption: the proportion (p) of visiting community pharmacy in this group is 50% because no published data was available the study area. Taking a Z value for 95% confidence interval (CI) (1.96) and marginal error (d) of 5%, the estimated sample size was calculated to be 384. Since the total number of households in Bonga town was less than 10000, the estimated sample was adjusted using sample size correction formula: S = (n×N)÷(n+N), where n and N represent the unadjusted sample size and total number of households (3956) respectively. The corrected sample size was 350 on which 10% was added for contingency, producing a final sample size of 385.

Sampling frame was created using sequential house numbers assinged to every household by the town’s authodities. Systematic random sampling was used to identify households from wich study participants were selected. An adult representative of each selected household was included in the study.

Data was collected using self-administered, semi-structured questionnaire. The questionnaire was divided in three sections the first of which focused on socio-demographic characteristics of the respondents. The second and the third sections covered the respondents’ past experience of visiting community pharmacies and their approval of provision of extended pharmacy services in community pharmacies respectively. Three college students who were on summer break were recruited to collect data by personally delivering questionnaires to respondents in selected households. The data collectors were given a training on data collection, including how to read the questionnaire to and record the responses of respondents that cannot read and write without providing extra explanation. The data collectors did not involve in further processing of data.

Letter of ethical approval was obtained from the ethical review board of the School of Pharmacy, University of Gondar. Terms and conditions of for participating in the study (including voluntary participation, option to skip any question they do not want to answer and avoidance of personal identifiers) were informed to all selected participants. Participants were asked to confirm their agreement to participate by filling the agreement form placed at the beginning of the questionnaire. Therefore, informed consent was obtained from all study subjects prior to administering the questionnaire.

The data was entered into IBM SPSS Statistics 25^®^ software. Results of the study were described by frequency, mean and standard deviation (SD). Binary logistic analysis was performed to identify potential associations between dependent and independent variables.

## Results

### Socio-demographic characteristics

Out of the 385 households initially selected by systematic random sampling, representatives of 29 households were not either available during data collection or willing to participate in the study. Thus, we were able to collect and analyze data from 356 households, producing a response rate of 92.5%. The majority (58.1%) of the study subjects were male in gender. The mean age of the participants was 34.7 ± 10.7 years with 46.2% of participants falling between 18 and 30 years of age. More than two-third (70.8%) of the participants were married, 44.1% have attended college and the majority earned monthly income not more than 3500 Ethiopian Birr (ETB) (Table 1).

**Table 1:** Socio-demographic characteristics of the study participants.

Variable	Frequency (%)
**Sex**	
Male	207 (58.1)
Female	149 (41.9)
**Age in years (n = 346)**^**a**^	
18–30	160(46.2)
31–45	144 (41.6)
46–60	32 (9.2)
> 60	10 (2.9)
Undisclosed	10
**Marital status**	
Single	86 (24.2)
Married	252 (70.8)
Divorced	11 (3.1)
Widowed	7 (2.0)
**Highest educational level attained**	
Illiterate	22 (6.2)
Only able to read and write-no formal education	11 (3.1)
Primary education (grades 1–8)	51 (14.3)
Secondary education (grades 9–12)	115 (32.3)
College education	157 (44.1)
**Employment status**	
Unemployed/stay-at-home parent	78 (29.1)
Self-employed	97 (27.3)
Civil servant	138 (38.8)
Business owner	19 (5.3)
Others^b^	24 (6.7%)
**Monthly income in ETB (n = 238)**^**a**^	
*≤* 2000	58 (24.4)
2001–3500	68 (28.6)
3500–5000	61 (25.6)
> 5000	51 (21.4)
Undisclosed	118

Note: ETB: Ethiopian Birr (at the time of data collection ETB/USD = 0.046)

^a^Not all participants provided information on the variables; percentages exclude missing cases

^b^Others: Student– 11, Private sector employee– 6; Pensioner– 4, Law enforcement officer– 2; Priest– 1

### Extent of use of extended pharmacy services in community pharmacies

**Fig 1:**
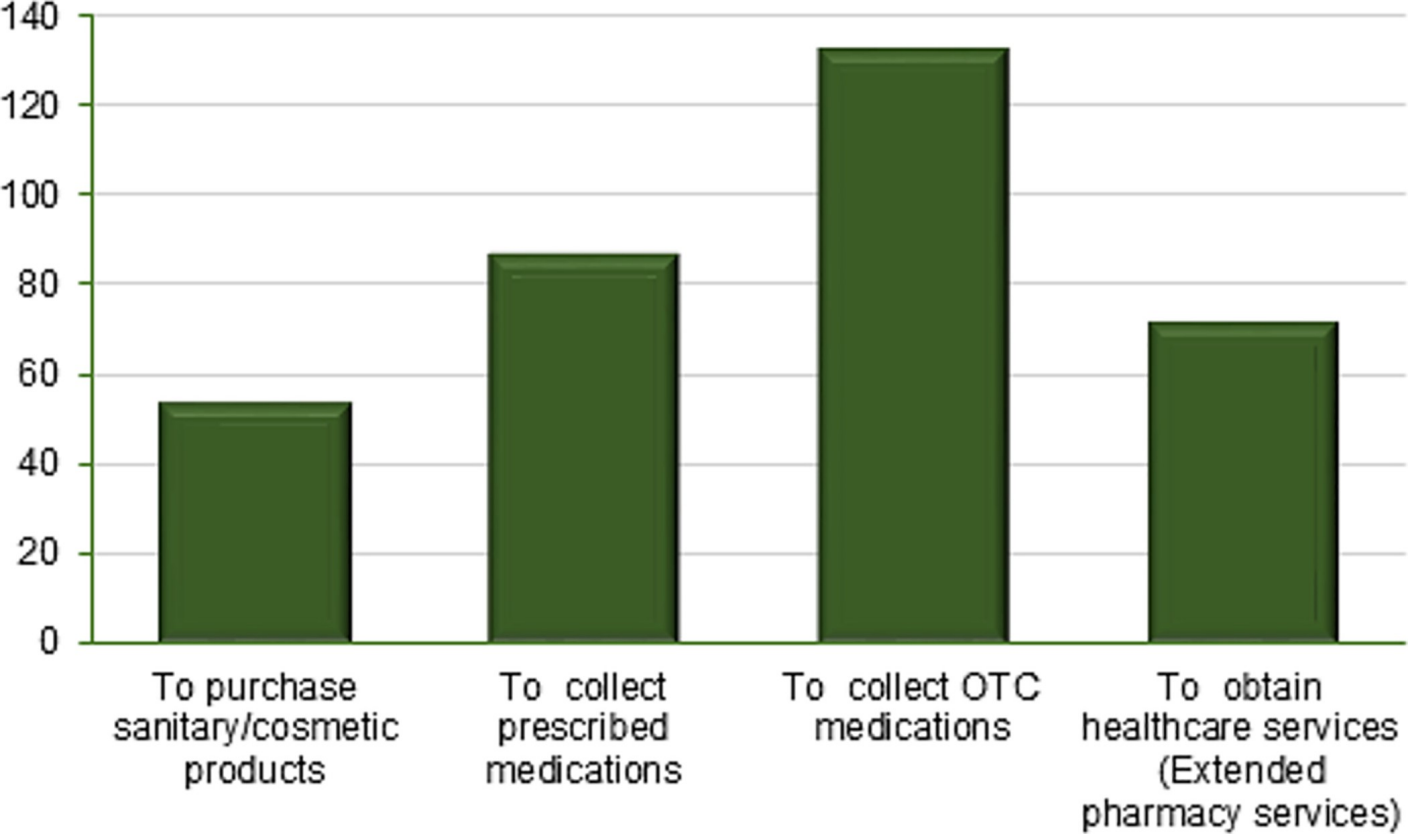
Reasons for visiting community pharmacy.

More than half of the respondents (58.4%) recalled visiting community pharmacy premises during the previous six months. The most common reason to visit community pharmacies was “to collect OTC medications” followed by “to collect prescription medications” (Fig 1).

**Fig 2:**
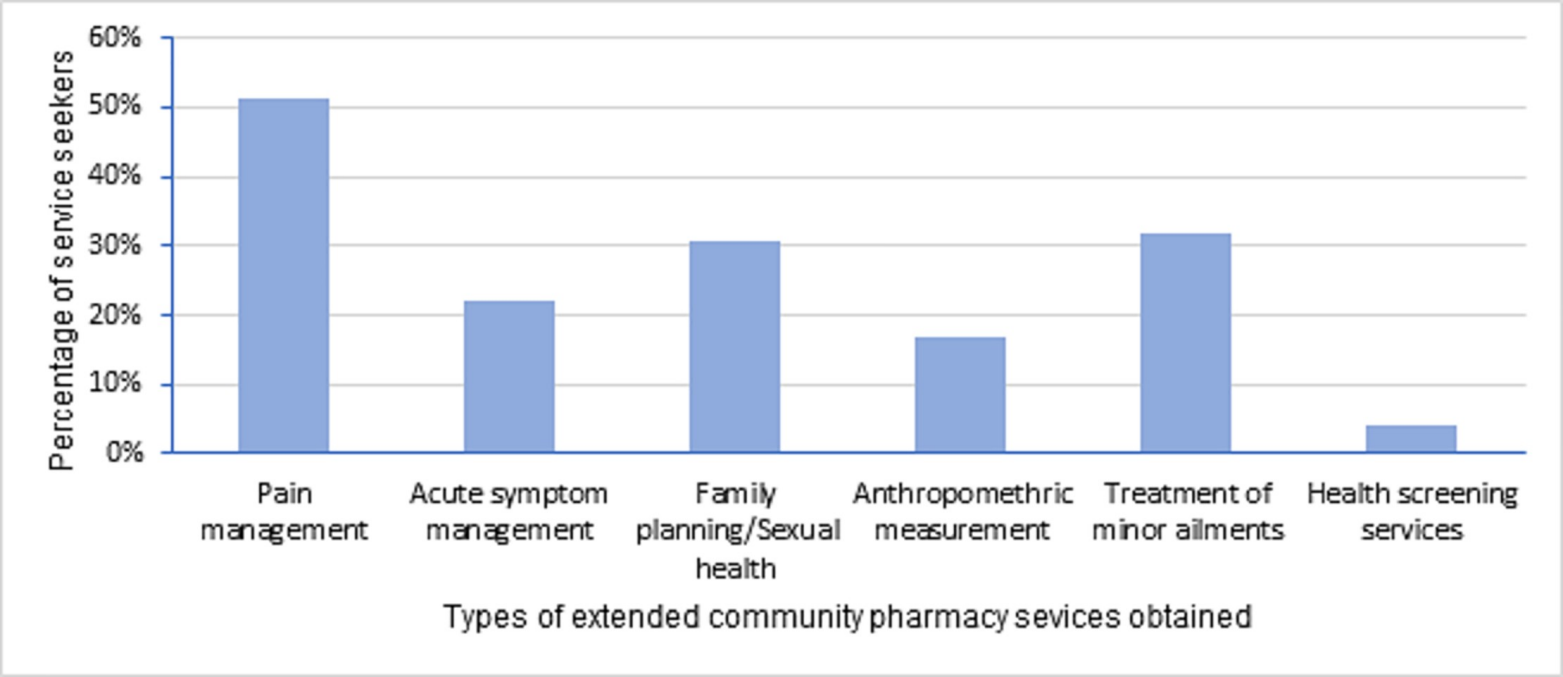
Types of extended pharmacy services obtained by community pharmacy visitors.

Out of the respondents who visited community pharmacies during the preceding six months, 72 (34.6%) did so to get extended pharmacy services. We summarized respondents’ descriptions of extended pharmacy services they got from community pharmacies in to seven categories of services as shown in Fig 2. Accordingly, pain management was the predominant service, having been obtained by more than half (51.4%) of these individuals.

### Extent of approval of provision of extended pharmacy services in community pharmacies

Asked about whether they approve the provision of extended pharmacy services in community pharmacies, 91.3% of the study subjects gave positive response. Regarding the types of extended pharmacy services, the majority of the respondents approved responding to symptoms (73.8%), and blood pressure, blood glucose and lipid testing (72.3%) to be provided at community pharmacies (Table 2).

**Table 2:** Extent of community’s approval of provision of different extended pharmacy services.

Extended pharmacy services	Extent of community’s approval	
	Yes [N (%)]	No [N (%)]
Anthropometric measurement	123 (37.8)	202 (62.2)
BP/sugar/lipid testing	235 (72.3)	90 (27.7)
Health screening	179 (55.1)	146 (44.9)
Responding to symptoms	240 (73.8)	85 (26.2)
Immunization	165 (50.8)	160 (49.2)

Abbreviation–BP, blood pressure

Respondents who approved the provision of extended pharmacy services in community pharmacies put forward several reasons for their approval. Absence of a need for appointment was stated by majority of the approving participants (72.0%) as a reason for their positive response followed by lesser waiting time as compared to primary care centers (66.8%) and proximity to their residence (45.8%).

Only 31 (8.7%) study subjects disapproved the practice of extended pharmacy services in community pharmacies. The main reasons reported for disapproving these services were inadequacy of pharmacists’ knowledge and skills (64.5%), and insufficiency of facilities in community pharmacy premises for such services (29.0%). The remaining 6.5% of the disapproving participants put forward concerns over pharmacists’ motivation/commitment to provide extended pharmacy services and legality of the practice as reasons for disapproving extended pharmacy services in community pharmacies.

### Factors associated with extent of use of extended pharmacy services

Binary logistic analysis was performed to identify the association between baseline variables and extent of use of extended pharmacy services. Accordingly, age, marital status and employment status were not determinants of extent of use of extended pharmacy services. On the other hand, college educated individuals [AOR = 19.37 (1.41, 265.57)] were 19.4 times more likely to have visited to get extended pharmacy services as compared to illiterate individuals whereas those who earned monthly income more than 5000 ETB (230 USD) [AOR = 3.61 (1.20, 10.83)] were 3.6 times more likely than those which earned 2000 ETB (92 USD) or less (Table 3).

**Table 3:** Binary logistic analysis of factors associated with extent of use of extended pharmacy services.

Variables	Visited CPs for EPS		COR (95% CI)	AOR (95% CI)
	Yes	No		
**Sex**				
Male	52	155	**2.16 (1.23, 3.81)**^**a**^	1.89 (0.83, 4.29)
Female	20	129	1.00	1.00
**Age**				
≤ 30	37	123	1.00	1.00
31–45	26	118	0.73 (0.42, 1.23)	0.76 (0.34, 1.66)
46–60	5	27	0.62 (0.22, 1.71)	1.01 (0.25, 4.14)
> 60	3	7	1.42 (0.35, 5.79)	2.64 (0.15, 46.32)
**Educational status**				
No modern education	2	20	1.00	1.00
Able to read and write	2	9	2.22 (0.27, 18.37)	3.24 (0.18, 57.35)
Primary education	2	49	0.41 (0.05, 3.10)	0.94 (0.06, 14.79)
Secondary education	7	108	0.65 (0.13, 3.35)	0.63 (0.04, 9.32)
College education	59	98	**6.02 (1.36, 26.69)**^**a**^	**19.37 (1.41, 265.57)**^**a**^
**Employment status**				
Unemployed/stay-at-home parent	7	71	1.00	1.00
Self-employed	12	85	1.43 (0.54, 3.83)	1.44 (0.31, 6.76)
Civil servant	39	99	**4.00 (1.70, 9.45)**^**a**^	0.49 (0.12, 2.11)
Business owner	8	11	**7.38 (2.23, 24.41)**^**a**^	2.34 (0.33, 16.68)
Other	6	18	**3.38 (1.01, 11.30)**^**a**^	0.91 (0.13, 6.46)
**Monthly income (ETB)**				
≤ 2000	10	48	1.00	1.00
2001–3500	12	56	1.03 (0.41, 2.59)	0.99 (0.33, 2.96)
3501–5000	18	43	2.01 (0.84, 4.82)	1.47 (0.51, 4.22)
> 5000	21	30	**3.36 (1.39, 8.10)**^**a**^	**3.61 (1.20, 10.83)**^**a**^

Abbreviations: AOR, adjusted odds ratio; CP, community pharmacy; CI, confidence interval; COR, crude odds ratio; EPS, extended pharmacy services; ETB, Ethiopian Birr (at the time of data collection ETB/USD = 0.046)

^a^Bold indicates significant at 95% confidence interval (p< 0.05)

## Discussion

The profession of pharmacy has undergone a rapid growth and development in the past decades [20]. In many countries, pharmacists in community pharmacies are involved in public health aspects of the community through screening and prevention of chronic diseases as well as management of minor ailments [11,21,22]. In Ethiopia, however, arguably most community pharmacies are confined to traditional practices of dispensing [23]. This study was launched with the objective of analyzing the extent of community’s use and approval of extended pharmacy services in community pharmacies.

Regarding the distribution of socio-demographic characteristics in our study participants, nearly 60% were male participants. College educated (44.1%) and civil servants (38.8%) were predominant regarding educational level and employment status respectively. These findings may be explained by the fact that our study enrolled a representative of each selected household and, taking in to account the social and cultural values of the community, men and the most educated household members are more likely represent their households. The predominance of civil servant participants is probably due to the fact that the role of private sector is negligible and government is the main employer in such small towns in Ethiopia.

In the present study, more than half of the community visited community pharmacy premises in the previous six months. The major reason for visiting a community pharmacy was to collect OTC medications. In line with the present study, a study conducted in Qatar showed that the major reason for visiting community pharmacies was to obtain OTC medications [24]. In contrast, studies done elsewhere showed that the community visited community pharmacies primarily for collecting prescription drugs [18,25–27]. Visiting community pharmacies mainly for purchasing of OTC medications could be an indication of individuals’ inclination of seeking symptom relief rather than treatment to the underlying cause of their ailments in our study setting. Lower accessibility of other healthcare facilities and prescribers may also be contributing factors. This argument can be supported by the fact that while collecting prescription medications was the second most common reason for visiting community pharmacy in our study, many studies showed that costumers visit community pharmacies primarily for collecting prescribed drugs [18,25–27].

The most common extended pharmacy service obtained by our study participants was pain management followed by treatment of minor ailments. Similarly, a study from Bosnia and Herzegovina showed that the majority of consumers would primarily seek advice from a community pharmacist for treatment of illnesses such as acne, minor skin lesions and constipation [25]. In contrast, study from Australia reported that more than 40% of community pharmacies gave asthma care, diabetes care, harm reduction with methadone, herbal medicines/nutritional supplement counselling, hypertension care and wound care [28]. Similarity with the findings of the Bosnia and Herzegovinian study may be related to comparable socio-economic status of the communities in the two studies whereas divergence from the Australian study can be explained partly by the difference in communities’ level of attention given to chronic diseases and partly by lower level of development of chronic disease management in community pharmacies in our study setting.

In this study, the extended pharmacy service that got highest approval was responding to symptoms (73.8%). Likewise, a study in Qatar reported that about 79% of subjects would like to see responding to minor ailments [24]. Other extended pharmacy services approved by our study participants include testing for blood pressure, serum glucose and lipid (72.3%), health screening (55.1%), immunization (50.8%) and anthropometric measurements (37.8%). Some similarity was seen with the finding of a study conducted in West Bank–Palestine which stated that 72.9% requested weight, height and temperature measurement; 87.5% blood glucose monitoring; 66.8% blood pressure monitoring and 59.1% demanded cholesterol level monitoring [29]. A study conducted in Addis Ababa, Ethiopia also showed that the majority of the respondents approved the introduction of the extended roles of community pharmacists, particularly screening for blood pressure (94.7%), blood cholesterol (89.5%) and blood glucose (91.0%), immunization, chronic disease management and counseling services (93.6%) [18]. A study done in Malaysia also showed that the services respondents would like to see in future were counseling on medication (54.4%) as well as diagnostic and screening services (48.9%) [30]. These findings show that despite geographical and socio-demographic diversities most communities strongly support the provision of extended pharmacy services at community pharmacies. Despite high rate of approval among the community in our study, certain elements of extended pharmacy services such as immunization and chronic disease follow-up are not yet legally permitted to be provided by community pharmacies in Ethiopia.

The present study showed that the major barrier for the provision of extended pharmacy services, as reported by our study subjects, was inadequacy of pharmacists’ knowledge and skills, followed by insufficiency of facilities in community pharmacies for such services. Similarly, pharmacists’ inadequate knowledge was also reported to be a barrier in studies conducted elsewhere [24,27]. Customers might have been skeptical of pharmacists’ knowledge and skill on provision of extended services because they have rarely seen pharmacists provide those services before. Nevertheless, the importance making sure that pharmacists are equipped with appropriate knowledge and skills for the provision of extended pharmacy services should not be overlooked.

The study showed that college educated individuals and those with monthly income more than 5000 ETB (230 USD) were more likely to have got extended pharmacy services at community pharmacies. College educated participants’ higher likelihood of visiting community pharmacies for extended services can be explained by their being more informed groups of the community regarding the healthcare services and acting accordingly to seek relatively new types of services delivered in community pharmacies. On the other hand, those with monthly income more than 5000 ETB, as they are economically more independent, can afford to visit community pharmacies more often and hence have more opportunity to obtain extended services at community pharmacies. Individuals’ financial status is an important determinant of access to healthcare services in Ethiopia since there is no functioning health insurance system in the country [31]. Participants’ proximity to community pharmacies was not included in the analysis as it was removed from the questionnaire after observation made by the investigators that all study participants were residents of a small town with evenly distributed community pharmacies in it and the proximity of each household to a community pharmacy would not be significantly different.

This study addressed issues related to a relatively new practice in Ethiopian healthcare system and covered an area often overlooked by public health researchers, hence producing completely new information on the topic of interest. However, the study has limitations too. For instance, the study is based on the responses of the study participants which make it prone to recall bias. Moreover, due to lack of similar studies done in other African countries, we were not able to compare our findings with these countries, which have comparable public health burden as well as similar socio-economic status. Despite its limitations, the findings of this study can be used as valuable inputs for other researchers and various stakeholders working on improving pharmacists’ role in public health.

## Conclusion

The extent of community’s use of extended pharmacy services at community pharmacies was found to be low. Nevertheless, majority of the study subjects approved the provision of extended pharmacy services at community pharmacies. Efforts to improve the practice of extended pharmacy service provision at community pharmacies should be made by all stake holders.

## Supporting information

S1 File Public’s perspective and extent of use of extended community pharmacy services in Southwest, Ethiopia. (SAV)

S2 File Questionnaire. (DOCX)
