# Supplementary material for: Community’s extent of use and approval of extended pharmacy services in community pharmacies in Southwest Ethiopia
Source: PLoS One. 2020 Apr 2;15(4):e0230863. doi: 10.1371/journal.pone.0230863 (PMC7117944; doi:10.1371/journal.pone.0230863)
Supplement: S2 File — (DOCX) [file pone.0230863.s002.docx]

**Study terms and conditions**

Hello,

We are conducting a designed to assess the extent of community’s use and approval of extended pharmacy services in Bonga town. You are one of the potential participants of the study randomly selected by the investigators. Your participation in this study is completely voluntary. The decision to participate or not to participate in the study only depends on your will. Your decision not to participate in this study will not in any way affect the service you get from those pharmacies or any other health institutions. Your name and address will not be recorded on this form and will never be used in connection with any of the information you provide. You can skip any question that you do not want to answer. However, we need your honest answers to the questions as it will help us get accurate data on community pharmacy services. The interview will take about 15-20 minutes.

We have included statements that summarize the above terms and conditions of the study at the beginning of the questionnaire. If you agree to participate in the study please place tic marks next to the statements to affirm your agreement. If any of those statements does not apply to you or you decide not to participate in the study, return the questionnaire to the data collector.

Thank you for your time.

**የጥናትና ምርምር ድንጋጌዎችና ሁኔታዎች**

ጤና ይስጥልኝ፣

በቦንጋ ከተማ የማህበረሰቡን በመድሀኒት ቤቶች የሚሰጡ ተጨማሪ የጠና ነክ አገልግሎቶች አጠቃቀም ደረጃና ለአገልግሎቶቹ መሰጠት ያለውን የድጋፍ መጠን ለመገምገም የሚያስችል ጥናትና ምርምር ቀርፀን በማካሄድ ላይ እንገኛለን፡፡ እርስዎ በተመራራማሪዎቹ በዘፈቀደ ከተመረጡት እጩ የምርምሩ ተሳታፊዎች መካከል አንዱ ነዎት፡፡ የሚሳተፉት ሙሉ በሙሉ በፍቃደኝነት ይሆናል፡፡ በጥናቱ መሳተፍዎ ወይም አለመሳተፈው የሚወሰነው በርሶ ፍላጎት ላይ ብቻ ነው፡፡ የርስዎ በጥናቱ ላይ ላለመሳተፍ መወሰን በምንም አይነት መልኩ ከእነዚህ መድሀኒት ቤቶች ወይም ከሌሎች የጤና ተቋማት ወደፊት በሚያገኙት አገልግሎት ላይ ተጽዕኖ አይኖረውም፡፡ ስምዎና አድራሻዎ በመጠይቁ ላይ የማይመዘገብ ከመሆነኑም በተጨማሪ በመጠይቁ ላይ ከሚሰጡት መረጃ ጋር በተያያዘ ፈፅሞ ጥቅም ላይ አይውልም፡፡ በመጠየቁ ላይ ካሉት ጥያቄዎች መመለስ የማይፈልጉት ካለ ትተው ማለፍ ይችላሉ፡፡ ነገር ግን፣ በመድሀኒት ቤቶች ስለሚሰጡ አገልግሎቶች ትክክለኛ መረጃ እንድናገኝ ይረዳን ዘንድ እውነተኛ ምላሽ እንዲሰጡን ያስፈልገናል፡፡

በመጠይቁ መጀመርያ ላይ ከላይ የተጠቀሱ የጥናቱን ድንጋጌዎችና ሁኔታዎች የሚያጠቃልሉ ንግርቶችን አካትተናል፡፡ በጥናቱ ለመሳተፍ የተስማሙ ከሆነ አባኮትን መስማማትዎን ለማረጋገጥ ከአረፍተነገሮቹ አጠገቡ ባሉት ሳጥኖች ውስጥ የጭረት ምልክት ያስቀምጡ፡፡ ከንግርቶቹ አንዱ የርሶን አቋም የማያመለክት ከሆነ ወይም በጥናቱ ላይ ላለመሳተፍ የወሰኑ ከሆነ መጠይቁን ለመረጃ ሰብሳቢው ይመልሱልን፡፡

ጊዜዎን ስለሰጡን እናመሰግኖታለን፡፡

Please make tic marks in the boxes if the following statements apply to you. Return the questionnaire to the data collector if at least of the statements do not apply to you.

I understand that participation in this study is completely voluntary. -------

I understand that my name and address will not be recorded. ----------------

I give my full consent to participate in this study. ------------------------------

1. **Socio-demographic characteristics**
2. Sex Male  Female
3. Age __________________ years
4. Highest educational level attained
   1. Illiterate ……………………………………………………….
   2. Only able to read and write …………………………………...
   3. Primary education (grade 1 – 8) ………………………………
   4. Secondary education (grade 9 – 12) ……….………………….
   5. College education ……………………………………………..
5. Occupation
   1. Unemployed/stay-at-home parent ………….…………………
   2. Self-employed ………………………………………………...
   3. Civil servant (non-military/security government employee) …
   4. Business owner ……………………….……………………….
   5. Other …………...……………………………………………...  Please specify ______
6. Marital status
   1. Single ……………………………………….…………………
   2. Married ………………………………………………………..
   3. Divorced ………………………………………………………
   4. Widowed ………………………………………....……………
7. Family’s monthly income in Ethiopian Birr _________________
8. **Use of extended pharmacy services**
9. Have you visited community pharmacy premises in the last 6 months?

Yes  No  If your answer is “No” please go to item number 10

1. If your answer to item number is “Yes”, for what purpose did you visit the community pharmacy? (Multiple response possible)
   1. To collect prescription medication ……………………………
   2. To collect over the counter (OTC) medication ….…………….
   3. To purchase sanitary/cosmetic products ………………………
   4. To get other healthcare services (extended pharmacy services)
2. If your response to item number 8 includes “To get other healthcare services (extended pharmacy services)”, describe the service you referred to as “healthcare service (extended pharmacy service)

________________________________________________________________________________________________________________________________________________________________________________________________________________

1. **Extent of approval of extended pharmacy services in community pharmacies**
2. Do you approve the provision of extended pharmacy services in community pharmacies?

Yes  No  (If your response is “No” please go directly to item number 13)

1. If your response to item number 10 is “Yes”, why do you think it is community pharmacies should provide extended pharmacy services? ______________________________________________________________________________________________________________________________________________________
2. If your response to item number 10 is “Yes”, which services should be provided by community pharmacies? (Multiple response possible)
   1. Anthropometric measurement …………………………..…..
   2. Blood pressure/ blood glucose/lipid measuring ….………….
   3. Health screening …………………………………….……….
   4. Responding to symptoms …………………………………....
   5. Immunization …………………………………………….…..
3. If your response to item number 10 is “No”, why do you think it is community pharmacies should not provide extended pharmacy services? ______________________________________________________________________________________________________________________________________________________

Thank you!

የሚከተሉት ዓረፍተነገሮች የርሶን አረዳድ የሚገልጹ መሆናቸውን ለማረጋገጥ ከጎናቸው በሚገኙት ሳጥኖች ውስጥ የጭረት ምልክት ያስቀምጡ፡፡ ከዓረፍተነገሮቹ ቢያንስ አንዱ የርሶን አረዳድ የማይወክል ከሆነ ወይም በጥናቱ ላይ ላለመሳተፍ የወሰኑ እንደሆነ መጠይቁን ለመረጃ ሰብሳቢው ይመልሱ፡፡

በዚህ ጥናት ላይ መሳትፍ ሙሉ በሙሉ በፍቃደኝነት ላይ የተመሰረተ መሆኑን ተረድቻለሁ፡፡ ------

ስሜና አድራሻዬ እንደማይመዘገቡ ተረድቻለሁ፡፡ ----------------------------------------------------------

በዚህ ጥናት ላይ ለመሳተፍ ሙሉ በሙሉ ፍቃደኛ መሆኔን አረጋግታለሁ፡፡ ---------------------------

1. **የሕበረተሰባዊና ሥነ-ህዝብ መረጃዎች**
2. ፆታ ወንድ  ሴት
3. እድሜ፡ __________________ ዓመት
4. የደረሱበት ከፈተኛው የትምህርት ደረጃ
   1. ምንም ዘመናዊ ትምህርት አልተማርኩም ……………………….
   2. ማንበብና መፃፍ እችላለሁ ……….……………………………….
   3. አንደኛ ደረጃ ትምህርት (1 – 8) ተምሬአለሁ ………………….
   4. ሁለተኛ ደረጃ ትምህርት (9 – 12) ተምሬአለሁ ……………....
   5. የኮሌጅ ትምህርት ተምሬአለሁ ……….………………………….
5. የተሰማሩበት የስራ አይነት
   1. ስራ የሌለው/የቤት እመቤት ……….……………………....…….
   2. የግል ስራ/የቀን ስራ ላይ የተሰማራ ……….…………………….
   3. የመንግስት ሰራተኛ (ወታደርና ፖሊስን አያካትትም) ………..….
   4. የንግድ ድርጅት ባለቤት ……….………………….……………..
   5. ሌላ እባኮት ይጥቀሱ ……….………………..…………………..
6. የጋብቻ ሁኔታ
   1. ያላገባ/ች ……….….…………………………………………….
   2. ያገባ/ች ………………………………………………………….
   3. የተፋታ/ች ……….……………………….…………………….
   4. ባለቤቱ/ቷ በሞት የተለየበት/ባት ……….……...……………….
7. የቤተሰብ ወርሃዊ ገቢ በብር __________________
8. **ተጨማሪ የመድሀኒት ቤት አገልግሎቶችን ስለመጠቀም**
9. ባለፉት ስድስት ወራት ውስጥ መድሀኒት ቤቶችን ገብኝተዋል?

አዎን  አልጎበኘሁም  (መልስዎ ‹‹አልጎበኘሁም›› ከሆነ ወደ ጥያቄ ቁ. 10 ያምሩ)

1. ለጥያቄ ቁጥር 7 የሰጡት መልስ ‹‹አዎን›› ከሆነ፣ ለምን ዓላማ ነው መድሀኒት ቤት የጎበኙት? (በርካታ ምላሾች መስጠት ይቻላል)

በሀኪም የታዘዙ መድሀኒቶችን ለመሸመት ……….……...……….

በሀኪም የማይታዘዙ መድሀኒቶችን ለመሸመት ……….……....….

የንፅህና መጠበቂያ/ የመዋቢያ ቁሳቁሶችን ለመሸመት …………….

ሌሎች የጤና አገልግሎቶችን/ተጨማሪ የመድሀኒት ቤት አገልግሎቶችን ለማግኘት ..

1. ለጥያቄ ቁጥር 8 የሰጡት መልስ ‹‹ሌሎች የጤና አገልግሎቶችን/ተጨማሪ የመድሀኒት ቤት አገልግሎቶችን ለማግኘት›› የሚለውን የሚያካትት ከሆነ፣ የተጠቀሟቸውን የጤና አገልግሎቶች ከዚህ በታች ይጥቀሱ

___________________________________________________________________________________________________________________________________________

1. **ተጨማሪ የመድሀኒት ቤት አገልግሎቶችን የመደገፍ ሁኔታ**
2. መድሀኒት ቤቶች ተጨማሪ የጤና ነክ አገልግሎቶችን መስጠታቸውን ይደግፋሉ?

አዎን  አልደግፍም  (መልስዎ ‹‹አልደግፍም›› ከሆነ እባኮትን በቀጥታ ወደ ጥያቄ ቁ. 13 ያምሩ)

1. ለጥያቄ ቁጥር 10 ‹‹አዎን›› የሚል መልስ ሰጥተው ከሆነ፣ መድሀኒት ቤቶች ተጨማሪ የጤና ነክ አገልግሎቶችን እንዲሰጡ የተስማሙበትን ምክንያት ከዚህ በታች ያብራሩ፡፡

___________________________________________________________________________________________________________________________________________

1. ለጥያቄ ቁጥር 10 ‹‹አዎን›› የሚል መልስ ሰጥተው ከሆነ፣ ከዚህ በታች ካሉት የትኞቹ አገልግሎቶች በመድሀኒት ቤቶች መሰጠታቸውን ይደግፋሉ? (በርካታ ምላሾች መስጠት ይቻላል)

የቁመትና ክብደት መለካት አገልግሎት ……….………….....….

የደም ግፊት/የስኳር መጠን/የሰውነት ቅባት መለካት …….....….

የጤና እክሎች ልየታ ……….…….....................................….

ለህመም ምልክቶች ተገቢ ህክምና መስጠት ……….……......….

የክትባት አገልግሎት መስጠት ……………………………....….

1. ለጥያቄ ቁጥር 10 ‹‹አልደግፍም›› የሚል መልስ ሰጥተው ከሆነ፣መድሀኒት ቤቶች ተጨማሪ የጤና ነክ አገልግሎቶችን መስጠታቸውን ያልደገፉበት ምክንያቶችን ከዚህ በታች ይዘርዝሩ፡፡

___________________________________________________________________________________________________________________________________________

እናመሰግናለን!
